# Evaluation of miR-34a Effect on *CCND1* mRNA Level and Sensitization of Breast Cancer Cell Lines to Paclitaxel

**DOI:** 10.29252/ibj.24.6.356

**Published:** 2020-06-02

**Authors:** Shiva Irani, Mahsa Paknejad, Masoud Soleimani, Azam Soleimani

**Affiliations:** 1 Department of Biology, Science and Research Branch, Islamic Azad University, Tehran, Iran;; 2 Department of Hematology, Faculty of Medical Sciences, Tarbiat Modares University, Tehran, Iran;; 3 Legal Medicine Research Center, Legal Medicine Organization, Tehran, Iran

**Keywords:** Breast cancer, Cyclin D1, Drug resistance, Paclitaxel, miR-34a

## Abstract

**Background:**

A growing body of literature has revealed the effective role of miR-34a, as a tumor suppressor and regulator of expression of multiple targets in tumorigenesis and cancer progression. This study aimed at evaluating the potential effects of miR-34a alone or in combination with paclitaxel on breast cancer cells.

**Methods:**

After miR-34a transduction by lentiviral vectors in two MCF-7 and MDA-MB-231 cell lines of breast cancer, effects of the elevated expression of miR-34a in the cell viability and the cell proliferation were determined using MTT assay in treated and untreated cells with paclitaxel. The mRNA level of the *CCND1 *gene was then measured in the two cell lines using the qRT-PCR assay. Finally, the influence of miR-34a and paclitaxel on apoptosis and cell cycle progression were examined by flow cytometry.

**Results:**

The *CCND1* mRNA expression levels were significantly down-regulated by overexpressed lentiviral miR-34a in MCF-7 and MDA-MB-231 cells. Combined treatment by miR-34a and paclitaxel reduced the cell viability and proliferation compared to single-drug treatment. In addition, the cell cycle arrest appeared at two phases by the combination of miR-34a and paclitaxel in MDA-MB-231 cells.

**Conclusion:**

Our results suggest that miR34a, in combination with paclitaxel, has a potential for decreasing the cell viability and proliferation. Moreover, it can reduce the expression of *CCND1* mRNA independent of the paclitaxel effect.

## INTRODUCTION

Breast cancer is the leading cause of death in women worldwide. The risk of breast cancer is about one in eight women so that 400,000 women die from this cancer every year^[^^1^^]^. MiRNAs are single-stranded noncoding RNAs with approximately 19–25 nucleotides in length^[^^2^^]^. Many studies have introduced miRNAs as a useful treatment response biomarker in various cancers^[^^3^^-^^7^^]^. The altered expression patterns of specific miRNAs and their effect on the target genes expression are associated with tumor progression and resistance to treatment^[^^8^^,^^9^^]^. Thus, specific miRNAs can cause resistance to chemotherapy and induce sensitivity to chemotherapy in cancer patients^[^^10^^-^^12^^]^.

The miR-34a mapped on chromosome 1p36.23 is widely found in all normal tissues, except for the lungs^[^^13^^]^. It has been reported that miR-34a is involved in the regulation of different cell activities such as tumor cell proliferation, invasion, and apoptosis, and it is significantly decreased in different tumor tissues, including colon cancer, prostate cancer, pancreatic, and breast cancer^[^^14^^-^^18^^]^. Specifically, the miR-34a binds to the 3'-UTR of *CCND1*,* CCNE2*,* CDK4*,* CDK6*,* N-MYC,* and so on. The *CCND1 *is one of the genes directly regulated by p53^[^^8^^,^^9^^]^. The* CCND1* is located in chromosome 11q13 and acts as a cell cycle regulatory protein mediating G1-to-S phase transition^[^^19^^]^. Overexpression of its protein has been reported in 42–80% of primary breast tumors and breast cancer cell lines^[^^20^^-^^24^^]^. However, the *CCND1* gene amplification has been observed in approximately 30% of primary breast tumors, and it may be associated with poor survival^[^^22^^,^^25^^]^.

The U.S. Food and Drug Administration approved paclitaxel for breast cancer treatment in 1994. The mechanism of action of this anti-tumor drug is to interfere with the normal function of the microtubules and induce the polymerization of microtubules. It also disrupts normal cell functions in mitosis and causes apoptosis^[^^26^^]^. Paclitaxel interferes with varied signal pathways and inflammatory mediators such as NFkB and interleukins, leading to inflammatory responses. Clinical failure of the paclitaxel in its long-term use can be due to its upregulation of host inflammatory responses. Therefore, precise timing is essential for the therapeutic function of this drug^[^^26^^-^^30^^]^. Unfortunately, many types of cancer are still resistant to chemotherapy. The mechanism of resistance to anticancer drugs is widely investigated, but our knowledge of it is not complete yet. A number of studies have reported the effective role of miRNAs in drug resistance^[^^30^^-^^32^^]^. Breast cancer that represents about 31% of all cancers in women has a high rate of drug resistance^[^^12^^,^^33^^]^. Therefore, it seems that miRNAs functions can provide hope for their clinical applications in the field of chemotherapy. 

Although numerous studies have investigated the effects of miR-34a on various cancers, few studies have addressed the combined function of miR-34a and chemotherapy in breast cancer. Hence, this study is aimed at evaluating the effects of miR-34a in combination with paclitaxel on the cell lines of breast cancer. 

## MATERIALS AND METHODS

**Cell culture**

The cell lines, MCF-7: Luminal A (ER^+^/PR^+^/HER2^-^), wild-type P53, MDA-MB-231 (triple-negative (ER^-^/PR^-^/HER2^-^)-mutant P53, and HEK 293T (human embryonic kidney cell line), were procured from Pasteur Institute of Iran, Tehran. Bacteria DH5α (containing PAX and pMD plasmids) and STBL4 (containing transfer plasmid pLEX-JRed-TurboGFP has-miR-34a gene) were supplied from Stem Cell Technology Research Center, Tehran, Iran.

**Plasmids**

***pLEX-JRed-TurboGFP***

This vector contains the genes required for the stable expression of miR-34a in eukaryotic cells and has several components: (1) a gene for the expression of GFP in transduced cells, which allows for the observation of cells with a blue filter in green fluorescence microscopy; (2) kanamycin resistance gene for the screening of bacterial cells containing this specific plasmid; (3) the puromycin resistance gene used to screen eukaryotic cells infected with the viral vector. This SV40 promoter is located before the gene for the overexpression of eukaryotic cells; (4) an origin of replication for proliferation in bacterial cells; (5) a CMV promoter inserted before the gene to increase the transcription of the cloned gene of miR-34a.

***psPAX and PMD plasmids***

The psPAX plasmid carries two viral *gag* and *pol* genes and provides the proteins necessary for the construction and packaging of the virus. PMD plasmid contains the genes required for encoding the viral envelope glycoproteins called VSV-G. Both plasmids have the ampicillin resistance gene. Simultaneous transfection of the three above-mentioned plasmids into HEK 293T cell line produces the lentivirus that contains our target gene. These viruses exit from the cell by budding and released to the culture medium.

***Propagation of plasmids***

DH5α bacteria (20 µl) containing PAX and PMD plasmids or STBL4 containing transfer plasmid (pLEX-JRed-TurboGFP has-miR-34a gene) were incubated in 3-5 ml of liquid Luria broth medium at 37 ºC for 4-5 hours. Then for monitoring and obtaining a high-density cell growth, 300-400 µL of this medium was added to a liquid Luria broth culture medium containing the selective antibiotic and incubated at 37 ºC for 16 hours. All three plasmids from bacteria) DH5α and STBL4) were extracted from the grown bacteria using MN-Midi plus kit (Germany) based on the manufacturer’s protocol. The quality of the extracted plasmids was evaluated by spectrophotometry and gel electrophoresis. 

**Production of lentiviral vector**

**Fig. 1:**
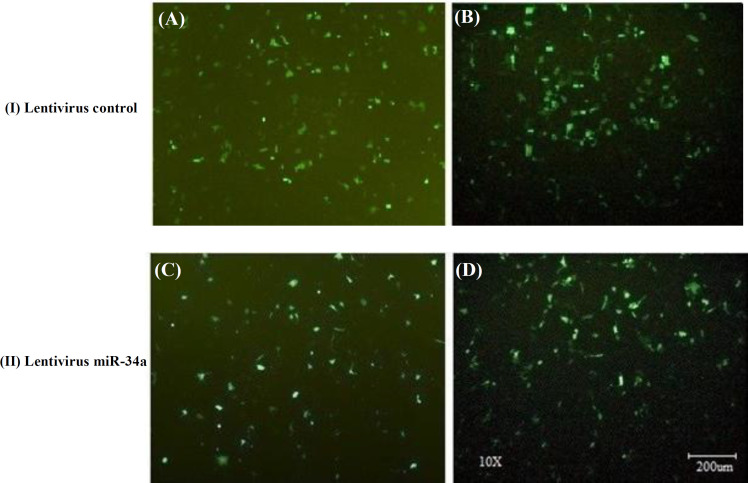
Fluorescent microscope image of GFP-positive transduced cell lines MCF-7 (A and C) and MDA-MB-231 (B and D) with scramble (control) (I) and lentivirus miR-34a (II)

Twenty-four hours before transfection, 2-3 × 10^6^ HEK-293T cells were cultured in a 25-cm flask containing Roswell Park Memorial Institute medium supplemented with10% FBS (Gibco, Germany) and incubated at 37 °C for 24 h. For the production of a lentiviral vector, three (PAX, PMD, and transfer) plasmids were extracted, and a lentiviral control (scramble) was simultaneously inserted into HEK-293T cells using the calcium phosphate transfection method. After virus budding from HEK-293T cells, the lentiviral vector was collected and condensed by the polyethylene glycol solution. An empty plasmid was used as the negative control lentivirus expressing the miR-34a gene (Fig. 1). The advantage of using the control virus is that the recombinant virus is influenced by the target cells to assess whether the changes in the cell are due to the transmitted gene or due to the virus itself. Thus, the GFP marker gene was used to determine the transfection rate. The HEK-293T cells were incubated with different concentrations of lentiviral vector, and after 72 hours, the expression of GFP reporter protein was examined by fluorescence microscopy, and the percentage of GFP-positive cells was evaluated by flow cytometry. The tests can only be performed if ≥70% of the cells are GFP-positive Since the virus genome contains the puromycin resistance gene, transduced cells were screened from other cells by antibiotic selection.

**Transduction of target cells and evaluation of paclitaxel drug effect**

Based on previous studies on breast cancer cell lines, including MCF-7 and MDA-MB-231, the multiplicity of infection (about 3–5) was used for transduction by a lentiviral vector. The metabolic activity of cells was measured by MTT method. The MCF-7 and MDA-MB-231 cells were treated with paclitaxel at the concentrations of 10, 30, 50, 70, 90, and 110 ng/ml, and then IC_50_ of the paclitaxel and cell viability were evaluated by MTT assay after 24, 48, and 72 h. All reactions were performed in triplicate. 

**RNA extraction**

Speciﬁc qPCR primers were supplied by Stem Cell Technology Research Center (Table 1) and veriﬁed in the BLAST website (https://blast.ncbi.nlm.nih.gov/ Blast.cgi). After transduction, extraction of miR-34a and its reference gene were performed in accordance with the manufacturer’s protocol of RNeasy Protect Cell Mini Kit in three time points: 24, 48, and 72 hours (Qiagen, Germany). In addition, the total RNA was extracted by RNX-Plus (CinnaGen, Iran) for CCDN1 and its reference gene. After 24 h and confirming transduction by fluorescence microscopy, the effective dose of paclitaxel was added to the transduced cells. Then the extraction of miR-34a and total RNA were performed separately to evaluate the effect of the paclitaxel on cells after 24 hours.

**Table 1:** Primers used in the study

** Sequences**	**Gene**
F: 5ʹ-ATG CCT GCC GTG TGA AC-3ʹ	*β* *2M*
R: 5ʹ-ATC TTC AAA CCT CCA TGA TG-3ʹ	*β* *2M*
	
F: 5ʹ-AGG GTG GCA GTG TCT TAG C-3ʹ	*miR-34a*
R: 5ʹ-GAG CAG GGT CCG AGG T-3ʹ	*miR-34a*
	
F: 5ʹ-ATC ACT GTA AAA CCG TTC CA-3ʹ	*SNORD-47*
R: 5ʹ-GAG CAG GGT CCG AGG T-3ʹ	*SNORD-47*
	
F: 5ʹ-CCC TCG GTG TCC TAC TTC AAA TG-3ʹ	*CCND1*
R: 5ʹ-CCT CCT CGC ACT TCT GTT CC-3ʹ	*CCND1*

F, forward; R,reverse

**cDNA synthesis and qRT-PCR**

Because of the short length, miR-34a and its reference gene (miR-34a and SNORD47) were first amplified by stem-loop RT-specific primers and then by M-MLV Reverse Transcriptase (Promega, USA). The random hexamers were used to synthesize the cDNA from *CCDN1* and *β2M *genes. Real-time PCR was performed using SYBR Green PCR Master Mix (Takara, Japan). Finally, the relative expression was performed using 2^–ΔΔCT ^method in Rest 2009 software.

**Cell cycle analysis and flow cytometry**

After preparation, the cells were stained with propidium iodide at 37 °C for 1 h. The cell cycle was then evaluated by flow cytometry, and the obtained data were analyzed using FlowJo 7.6.1 program.

**Statistical analysis**

All tests were repeated at least three times, and the results of tests were expressed as mean ± SD. Student’s *t*-test and ANOVA were used to compare the mean values obtained from the test results. All data were analyzed using SPSS 19.0, and *p* < 0.05 was considered as statistically significant.

## RESULTS

**Quality and quantity of extracted plasmid DNA**

**Fig. 2:**
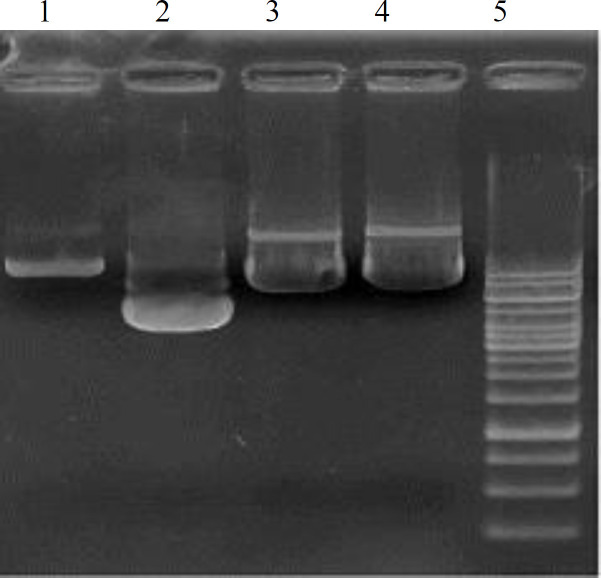
Electrophoresis of plasmids on 1% agarose gel. The lanes represent DNA extracted from psPAX2Sal (lane 1), pMD2.G (lane 2), pLEX-Blank (lane 3), pLEX miR-34a (lane 4), and 1 kb ladder (lane 5).

The bands obtained by electrophoresis showed that the extracted plasmids were of high quality )Fig. 2). In addition, the extracted DNA using spectrophotometer indicated a high concentration of 500 ng/µl with the purity of 1-2.

**Transfection of target cells**

**Fig. 3:**
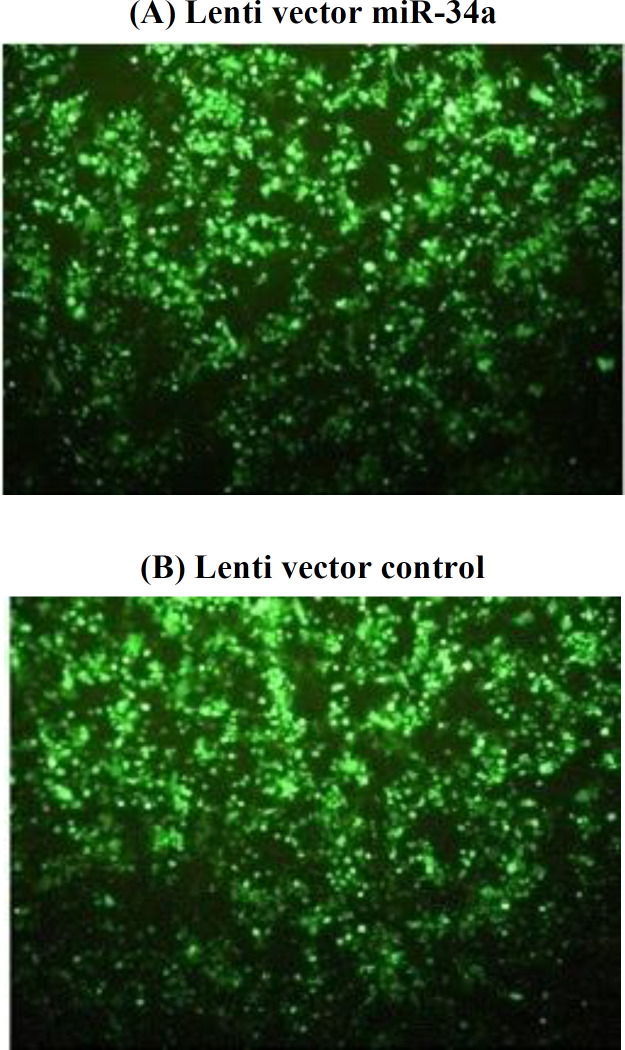
Fluorescent microscope image of transfection results with calcium phosphate assay and lentivirus production. HEK293T cells containing GFP marker was transfected with two plasmids: miR-34a (A) and control (B).

In transfection of HEK293TT cell line, the large number of green-fluorescent cells expressing the transfer vector (with GFP marker) indicates the high efficiency of transfection in both groups (plasmid containing miR-34a and control plasmid), approximately 80% transfection (Fig. 3). MCF-7 and MDA-MB-231 cells also exhibited GFP expression, indicating the successful transfection process (Fig. 1). Since the GFP marker is located just behind the target gene (miR-34a), these cell lines will express a high level of this miRNA. 

**Paclitaxel’s** **IC**_50_ **in MCF-7** **and** **MDA-MB-231** **cell lines **

**Fig. 4:**
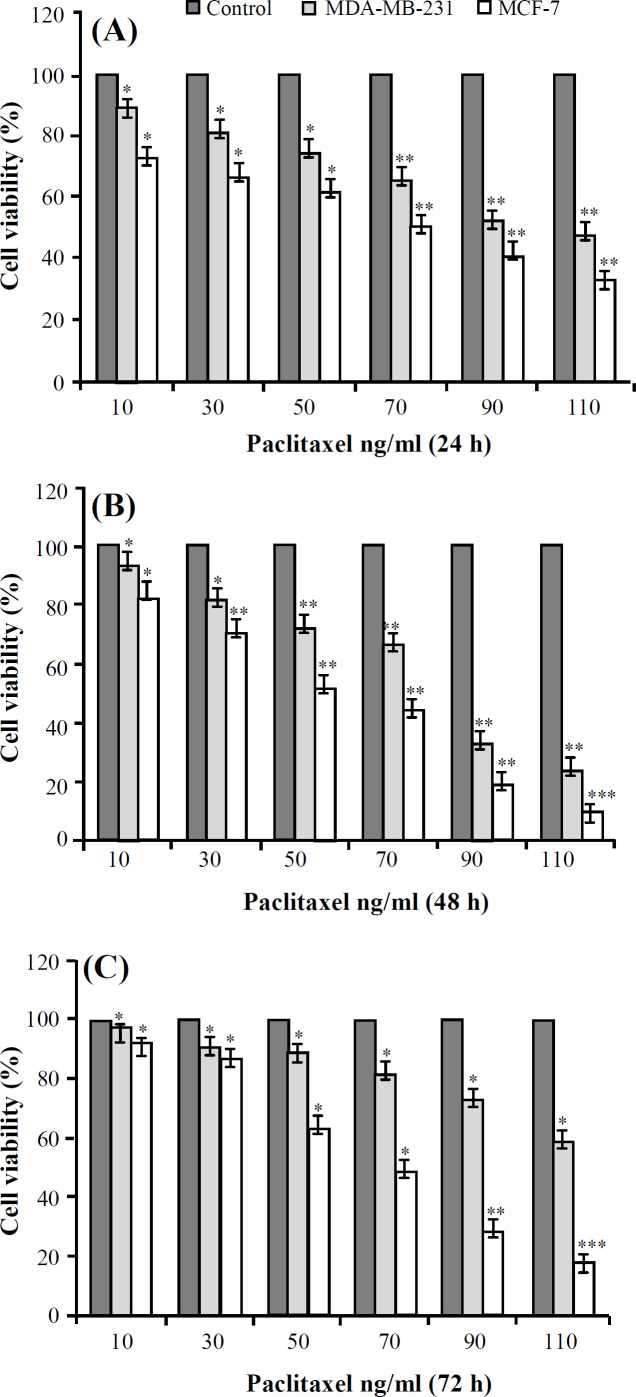
Paclitaxel’s IC_50_ in miR-34a in MDA-MB-23 1and MCF7 cells for (a) 24, (b) 48, and (c) 72 hours after treatment with both cell lines, based on changes in the concentration of the paclitaxel drug (^*^p ≤ 0.001, ^**^p ≤ 0.05, and ^***^p ≤ 0.01).

Based on Figure 4, after 24 hours, about one-half of the cells were destroyed; however, after 48 hours an imbalance appeared between the high dose and the low dose of the drug in reducing metabolic activity of the cells. According to the results, the effective doses of the drug for the MCF-7 and the MDA-MB-231 cell lines were 70 ng/ml and 90 ng/ml, respectively. Overall, the effective dose of the drug was considered after 24 hours of exposure.

**Comparison of cell viability and proliferation**

**Fig. 5:**
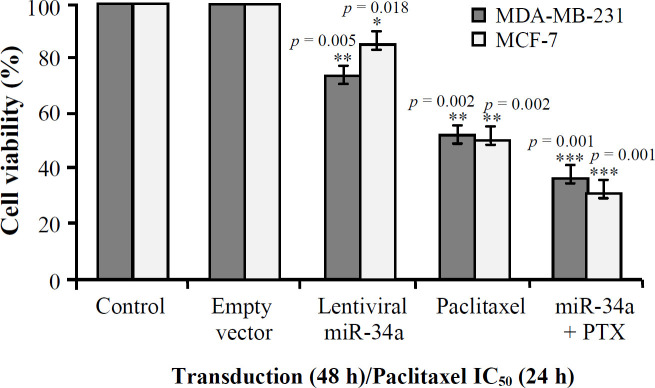
MTT assay of miR-34a (48 h), paclitaxel (PTX; 24 h), and miR-34a + PTX (^*^p ≤ 0.001, ^**^p ≤ 0.05, and ^***^p ≤ 0.01).

Based on Figure 5, in both MCF7 and MDA-MB-231 cell lines, cell viability and proliferation significantly decreased in the group treated with lentiviral miR-34a and paclitaxel compared to single treatments (*p* = 0.001). 

**Expression changes of miR-34a and** ***CCND1*** **genes**

**Fig. 6:**
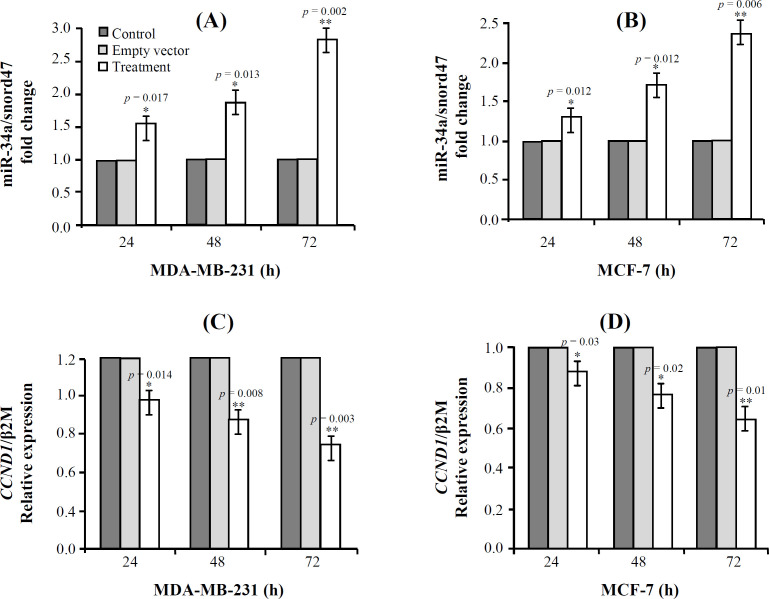
Expression of miR-34a and *CCND1* genes in MDA-MB-231 (A and C) and MCF-7 (B and D) cells (^*^p ≤ 0.05 and ^**^p ≤ 0.01).

Figure 6 shows that 72 h after transduction, miR-34a expression increases about 2.8fold (*p* = 0.002) in MDA-MB-231 cells, and there is about 40% (*p* = 0.003) decrease in the expression level of *CCND1* mRNA compared to the control. Moreover, in MCF-7 cells, miR-34a expression increases to about 2.2fold (*p* = 0.006) at 72 h after transduction, and the expression level of *CCND1* mRNA decreases about 30% (*p* = 0.01) compared to control (*p* > 0.05). 

**Expression of miR-34a and** ***CCND1*** **after treatment with paclitaxel**

**Fig. 7:**
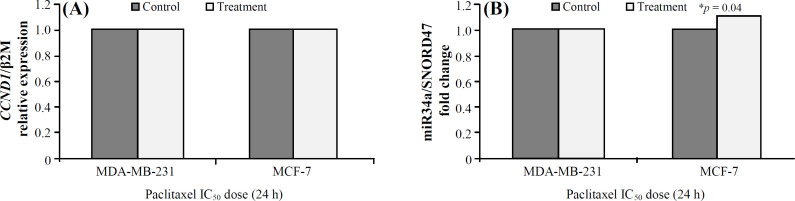
Expression of *CCND1* (A) and miR-34a (B) after treatment with paclitaxel (^*^p <0.05)

In MCF-7 cell line, the expression level of miR-34a increased significantly about 1.1fold (*p* = 0.04) after treatment with paclitaxel; however, there was no significant difference in the *CCND1* gene expression in both cell lines (*p* > 0.05; Fig. 7). 

**Treatment with paclitaxel and cell cycle analysis**

**Fig. 8:**
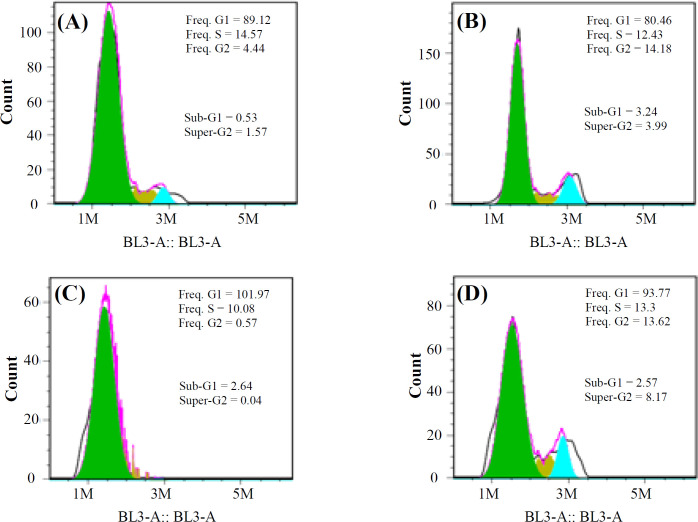
MDA-MB-231 cell cycle analysis by flow cytometry in three different treatments. (A) control samples, (B) paclitaxel, (C) miR-34a, and (D) miR-34a + paclitaxel

Following treatment of cells with the combination of paclitaxel and miR-34a, the MDA-MB-231 cell line was arrested more in the Sub-G1 phase, as compared to the control sample. Cells transduced only with miR-34a lentivirus were arrested more in the Sub-G1 phase. When cells received the combined treatment (paclitaxel and miR34a), the peaks of both G1 and G2 phases increased, as compared to the control sample (Fig. 8).

## DISCUSSION

Breast cancer, which accounts for about 31% of all cancers in women, shows a high percentage of drug resistance^[^^34^^]^. Several mechanisms are *associated *with cancer drug resistance, including decreased antitumor drug uptake, modified drug targets, altered cell cycle checkpoints, or increased DNA damage repair^[^^35^^]^. Emerging studies have highlighted the role of miRNAs in drug resistance through the modulation of genes involved in cell proliferation, cell cycle, and apoptosis^[^^35^^]^.

Paclitaxel, a drug with relatively low toxicity, has widely been applied in combination with other medications, to treat various human cancers such as breast cancer^[^^27^^,^^30^^,^^36^^,^^37^^]^. Nonetheless, the use of paclitaxel is limited due to serious side effects and drug resistance^[^^36^^,^^37^^]^. Many studies have supported the association between the downregulation of miRNAs in various cancers, including breast cancer. In addition, it has been reported that the dysregulation of different miRNAs can modulate sensitivity to paclitaxel in breast cancer^[^^34^^-^^37^^]^. However, the effect of miR-34a on the sensitivity of breast cancer cells to paclitaxel has not yet been reported.

The effect of increased expression of miR-34a alone or in combination with paclitaxel on the cell viability and cell proliferation in breast cancer cell lines was examined. There was a significantly more decrease in the cell viability and proliferation in the group treated by a combination of lentiviral miR-34a and paclitaxel compared to the cells treated by miR-34a or paclitaxel alone (*p *= 0.001). The miR-34a overexpression in the MCF-7 (*p *= 0.006) and MDA-MB-231(*p *= 0.002) breast cancer cells was significantly associated with the decreased mRNA expression of *CCND1* gene *in vitro*. Besides, no changes were observed in *CCND1 *expression levels for the combined treatment of miR-34a and paclitaxel in either cell lines (Fig. 7). This result is similar to our previous report on SW480 CRC and another study that showed paclitaxel probably inhibits only the depolymerization of microtubules, and it is not involved in transcriptional machinery^[^^38^^,^^39^^]^.

However, Kastl *et al.*^[^^40^^]^ reported that the direct interaction of miR-34a with *BCL-2* and *CCND1* is correlated with the drug resistance of docetaxel in breast cancer, which is likely due to the different mechanisms of the drugs. In this study, cell cycle analysis showed that after treatment by paclitaxel and miR-34a, the peak of *cell *cycle *arrest* in G2 phase increased in MDA-MB-231 cell line compared to treatment by miR-34a or paclitaxel alone (Fig. 8). 

The observed reduction in *CCND1* mRNA expression was independent of the combined therapeutic effect of miR-34a and paclitaxel, which explains that the combination of miR-34a and paclitaxel on the cell cycle does not depend on the direct role of *CCND1*. Understanding the mechanism of function of these two genes on the cell cycle needs further investigation.

Overall, following treatment of breast cancer cell lines with miR-34a alone or in combination with paclitaxel, significant decrease in the cell viability and proliferation was observed. Moreover, induction of miR-34a expression by increasing the sensitivity of cancer cells to chemotherapy and reducing *CCND1* mRNA has entered two simultaneous positive effects on breast cancer. Therefore, reactivating miR-34a in patients with breast cancer may be a chance to reduce the effect of paclitaxel drug resistance, though there is a need for more studies in this area.

## CONFLICT OF INTEREST.

None declared.
